# Combining Social Science and Environmental Health Research for Community Engagement

**DOI:** 10.3390/ijerph16183483

**Published:** 2019-09-19

**Authors:** Alissa Cordner, Grace Poudrier, Jesse DiValli, Phil Brown

**Affiliations:** 1 Sociology Department, Whitman College, Walla Walla, WA 99362, USA; 2 Department of Sociology and Anthropology, Northeastern University, Boston, MA 02115, USA; poudrier.g@husky.neu.edu (G.P.); divalli.je@husky.neu.edu (J.D.); p.brown@northeastern.edu (P.B.); 3 Department of Health Sciences, Northeastern University, Boston, MA 02115, USA

**Keywords:** social science-environmental health collaboration, community-based participatory research, community-engaged research, civic science, environmental justice, ethnography, chemical legacy, Superfund

## Abstract

Social science-environmental health (SS-EH) research takes many structural forms and contributes to a wide variety of topical areas. In this article we discuss the general nature of SS-EH contributions and offer a new typology of SS-EH practice that situates this type of research in a larger transdisciplinary sensibility: (1) environmental health science influenced by social science; (2) social science studies of environmental health; and (3) social science-environmental health collaborations. We describe examples from our own and others’ work and we discuss the central role that research centers, training programs, and conferences play in furthering SS-EH research. We argue that the third form of SS-EH research, SS-EH collaborations, offers the greatest potential for improving public and environmental health, though such collaborations come with important challenges and demand constant reflexivity on the part of researchers.

## 1. Introduction

While the social sciences are often seen as a disciplinary world removed from environmental health science, there is a long historical tradition of merging some social science into environmental health research. Furthermore, environmental and medical sociologists have long depended on (and critiqued) contributions from natural science and biomedical fields. In the last two decades, a strong framework has emerged that explicitly integrates social science and environmental health science, sparking a network of scholars and community members who use combined science to improve individual, local, and global environmental health [1,2].

Contemporary social science-environmental health (SS-EH) collaborations are highly engaged in community environmental health settings and operate on multiple levels. While SS-EH research does not necessarily involve community collaboration, SS-EH and community-engaged research approaches have nourished each other and are often part of the same projects. Most of these collaborations incorporate an explicit environmental justice (EJ) approach that recognizes the disproportionate exposure to environmental hazards and pollution experienced by people of color, low income, immigrant, and other marginalized populations, even when researchers are not directly engaged with EJ community groups.

In this article we address the nature of SS-EH contributions and propose a three-part typology to situate different SS-EH practices within a larger transdisciplinary sensibility: (1) environmental health science as influenced by social science; (2) social science studies of environmental health; and (3) social science-environmental health (SS-EH) collaborations. We describe examples from our own and others’ work, and we discuss the central role of research centers, training programs, and conferences in furthering SS-EH research. We argue that SS-EH collaborations (the third form of SS-EH research in our typology) offer the greatest potential for improving public and environmental health. At the same time, we emphasize the ways in which the challenges of SS-EH collaborations demand constant reflexivity on the part of researchers. This typology provides a way for scholars and practitioners across the field to envision the diverse possibilities for SS-EH collaboration, identify the resources and types of training necessary and useful for different forms of SS-EH research, and reflect on their practices in light of the diverse types.

## 2. Data and Methods 

This paper utilizes multiple data sources and is informed by our collective experience conducting SS-EH collaborative research over many years. First, we draw on our familiarity with the landscape of the SS-EH field from our vantage point as social scientists who research environmental health topics in collaboration with environmental health scientists. This includes our familiarity with literature by researchers and practitioners of the approaches we are investigating, as well as secondary analyses of such efforts (a comprehensive review of this literature is beyond the scope of this paper, but is available elsewhere (e.g., in [1])). Second, we offer insights gleaned from our attendance at many professional conferences over the years, including disciplinary conferences and grantee conferences of National Institute of Environmental Health Sciences (NIEHS) centers and programs (e.g., Superfund Research Program, Children’s Environmental Health Centers, Partnerships in Environmental Public Health, American Public Health Association, Society for Environmental Toxicology and Chemistry, American Sociological Association, and Society for the Social Study of Science). Finally, we draw from our experience practicing the three forms of collaboration identified in our typology in two research groups—The Contested Illnesses Research Group (CIRG) at Brown University (1999–2012) and the Social Science Environmental Health Research Institute (SSEHRI) at Northeastern University (2012–present)—which cumulatively provide a unique understanding of SS-EH interactions and collaborations. Our analysis is directly informed by our own work conducting the types of research collaborations herein discussed, and thus cites examples largely from our three decades of experience conducting community-engaged research.

## 3. Overall SS-EH Contributions

### Definition, Scope, and History of SS-EH

SS-EH involves close collaboration between social scientists and environmental health scientists and operates at multiple scales and through many different research forms. Sociologists, anthropologists, social psychologists, geographers, political scientists, science and technology studies (STS) scholars, historians, and economists have all been involved in social science approaches to environmental health. Much SS-EH work involves research teams or centers, especially for the collaboration type we discuss below in our typology. However, individual scholars can engage in SS-EH if they actively network and communicate with researchers on the other side of the dyad.

SS-EH is guided by a general set of underlying ethics and values. First, SS-EH is aligned with a precautionary approach to environmental health issues and recognizes the significant limitations in the current political-economic system regarding environmental health hazards, particularly in the United States. Research and action on environmental health is a necessary response to the negative actions of industry and the flawed or inadequate action of government regulatory agencies. Thus, the very act of identifying environmental health hazards as objects requiring study implies a strong statement about values. Additionally, this approach argues that solutions to environmental health problems should not be a matter of holding members of the public personally responsible for avoiding environmental hazards but rather a matter of demanding collective action and structural solutions to prevent and allocate responsibility for contamination.

Second, SS-EH research centers community-oriented research questions and practice, with a focus on marginalized and underrepresented groups and environmental justice (EJ) communities. The impetus for community engagement and SS-EH collaboration often stems directly from community activism and a continually expanding EJ orientation. This approach emphasizes the importance of deep involvement of affected communities in developing and carrying out the research questions that are important to them [3].

Third, SS-EH research includes a critique of objectivity as a value-driven approach to science. Instead of privileging expert-produced data as the primary source of legitimate knowledge, SS-EH takes seriously the centrality of lay knowledge and perceptions. This approach recognizes the ways in which industry and government have often hidden behind claims to objectivity when in fact they were defining a narrow range of acceptable knowledge.

NIEHS first began supporting the development of SS-EH with their Environmental Justice and community-based participatory research (CBPR) grant programs in the early 1990s, sometimes with grants that required a social scientist on the team [4]. Even when the involvement of social scientists was not required, the topics and settings funded by these programs often encouraged social scientist participation. Superfund Research Program Community Engagement Cores (CECs) and Research Translation Cores have been prominent vehicles for SS-EH. Similar forms of SS-EH work are undertaken in the CECs of other NIEHS centers, including at Children’s Environmental Health Centers, Deepwater Horizon Oil Spill Centers, Environmental Health Core Centers, and Breast Cancer and the Environment Research Program Centers.

However, not all SS-EH takes place within formal NIEHS-funded research efforts, and examples of other SS-EH research can be found in environmental sociology, environmental anthropology, STS, geography, and history. Independent environmental health science centers have long worked with social scientists. Two prominent examples are the Silent Spring Institute, founded by the Massachusetts Breast Cancer Coalition, and The Endocrine Disruption Exchange, founded by the late Theo Colborn. These centers maintain strong community collaborations and recognize the importance of the local community context in relation to the larger social context. Non-profit community-oriented organizations like the Public Laboratory for Open Science and Technology (discussed below) also practice SS-EH collaborations. Some conferences are notable for the amount of SS-EH work they incorporate into their sessions, including the Association of American Geographers, the Citizen Science Association, and the Society for Applied Anthropology.

Based on our evaluation of the SS-EH research field, we propose a preliminary typology of three broad categories, described in detail below: (1) environmental health science influenced by social science; (2) social science studies of environmental health problems; and (3) social science-environmental health collaborations. We argue that the third category invites the most complete integration of these fields, and thus has the greatest potential for innovative research methods and findings. However, we also note the value of other forms of SS-EH research, particularly since not all researchers will have the resources needed to have a fully collaborative SS-EH approach, and not all research questions and topics require the full collaborative model. Many factors influence which form of SS-EH research is used, including the training and experiences of researchers and research teams, levels of support from their department or institution, their prior links to community and scientific partners, their available career possibilities and choices, and the needs of affected communities with which they work. All varieties of SS-EH research serve community needs in some way and promote higher levels of engagement than traditional single-discipline scholarship.

## 4. Environmental Health Influenced by Social Science

Our first major category involves environmental health scientists who incorporate social science perspectives or ideas into their research. Many environmental health scientists provide resources to affected communities and larger national communities, and some have incorporated social science into their understanding of the field. Environmental health scientists at NIEHS point to the crucial role of social science research in demonstrating the importance of behavioral and social factors, such as poverty and relative income, the use of techniques such as network analysis to examine environmental disaster effects, and the application of Traditional Ecological Knowledge to EH overall [5]. They highlight social science contributions to understanding topics such as the social determinants of health and the role of social movements in pressing for public health prevention and amelioration [5].

Foundational environmental health researchers used social science to make sense of how powerful industries and inadequate regulatory systems created and exacerbated environmental health problems. Rachel Carson’s social scientific sensibility was apparent in her structural critique of the pesticide industry, which went far beyond the disciplinary boundaries of her training as a biologist [6]. The ecologist Barry Commoner was also trained as a biologist, and his scholarship and advocacy involved much broader arguments about population, sustainability, and capitalism; indeed, his 1971 *The Closing Circle* is widely read in environmental sociology graduate programs in the United States [7]. Similarly, the ecologist Sandra Steingraber, starting with her groundbreaking book *Living Downstream*, has always situated her life and natural science with a firm context of corporate practices, government policy, and community awareness [8].

This trend is also observable in recent environmental health research. For example, epidemiologist Steve Wing incorporated an environmental justice critique into his analysis of North Carolina hog farming and was forceful in vocally pointing this out at scientific conferences [9]. Epidemiologist Richard Clapp has a long tradition of writing about the political-economic context of communities affected by contamination and currently works with the Lowell Center for Sustainable Production, a transdisciplinary research center focused on green chemistry and reconceiving production to achieve a cancer-free economy [10].

This form of SS-EH is common when EH researchers want to move outside of the EH silo and speak to a broader audience, particularly when working on public health issues of pressing importance. For example, the Center for the Health Assessment of Mothers and Children of Salinas (CHAMACOS) study, led by neuropsychologist and epidemiologist Brenda Eskenazi, investigates the transgenerational impacts of pesticide and other environmental exposures on farmworkers and their families, with a high degree of long-term engagement with their cohort [11]. NIEHS Superfund Research Program (SRP) Centers have institutionalized an engaged form of environmental health science through their community outreach requirement. Successful SRPs engage in community outreach that goes far beyond the translation of research findings to impacted communities. SRP CECs “enhance knowledge exchange and… support the needs of communities impacted by hazardous-waste sites” by helping communities access information, answering scientific questions and providing scientific expertise, and partnering directly with tribes [12].

For example, Brown University’s SRP CEC helped local activists found the Environmental Justice League of Rhode Island in 2007 [13]. As another example, the Community Outreach and Engagement Core (COEC) at the University of Rochester Environmental Health Core Center has worked for nearly two decades on local lead poisoning prevention efforts, including research into how children’s blood lead levels affected development at lower levels than were previously known. These researchers emphasize the importance of social science concepts and methods, including policy analysis, economic projections, survey research, and qualitative methods [14]. Other SRP COECs have had similar involvement with social science approaches. One such example is Columbia University, where the COEC deputy director was a sociologist who assisted with survey design, focus groups, and interviewing in an Environmental Protection Agency (EPA) Community Action for a Renewed Environment (CARE) program [14].

## 5. Social Science Studies of Environmental Health

Our second major category includes research in which social scientists investigate environmental health crises, exposures, contamination, disasters, and policies [2]. This category includes the political, economic, legal, cultural, and social dimensions of environmental health problems, the social dynamics of illness and exposure contestation, and the unequal power relations between polluters, communities, government officials and regulators, scientific experts, and activists in conflicts over environmental health. Key topics include the social-psychological, political, community, and racial and class components of environmental hazards and contamination. Social scientists also made significant contributions by pursuing ethical approaches to EH issues, especially around broadly expanded right-to-know approaches for research participants, and around ethical and social implications such as privacy, safety, community participation, and extension of human subject’s protection from individuals to communities. Social scientists studying environmental or health problems often develop advanced levels of technical knowledge to understand scientific controversies and policy issues.

### 5.1. Case Studies of Contaminated Communities

Some of the earliest contributions of social science to environmental health were ethnographic studies of contaminated communities (see a review in [1]). These ethnographies detailed the processes of exposure and building of knowledge and awareness of significant exposures, becoming central motivators of additional scholarship within the social science of EH. They include Erikson’s examination of the destruction of Buffalo Creek, KY by the floodwaters of a coal slurry whose dam broke [15]; Levine’s study of buried toxic wastes that resurfaced in the middle of the residential neighborhood at Love Canal in Niagara Falls, NY [16]; and Edelstein’s comparative analysis of water contamination in Legler, NJ and other communities [17]. Since these early studies, the field of inquiry for the social science of EH has ballooned, covering cases of discovery of and response to exposures, across the United States and internationally from highly varied social science fields, including sociology [18], political science [19], social psychology [20], and environmental justice [21].

These ethnographies of contaminated communities require some amount of EH knowledge by the researchers. Though they were not collaborations per se, they set the stage for understanding the broad social context of environmental contamination by offering the EH field a new set of factors to consider. These include the role of demographic factors in responses to contamination, the social-psychological experience of contamination, the importance of community responses in getting research and remediation done, the connections between regulatory failure and contamination episodes, the role of corporate malfeasance in hiding chemical data, the centrality of environmental justice factors dealing with race and class, and the importance of social capital and social networks in community response to toxic crises.

The methods employed by researchers have been as varied as the exposures described and social science fields from which they originated. In addition to the ethnographic designs described above, social scientists have brought other relevant qualitative research tools and methods to bear on environmental health topics, including interviews, participant observations of community meetings, and photo diaries. Quantitative methods employed by these researchers have included statistical analyses of surveys and vast environmental and economic data, geospatial information systems surveys, and economic and risk modeling of exposure-related harm. Social scientists with training in geospatial research methodologies have produced a huge body of research that has dramatically improved the research techniques used to examine how exposure to pollutants impacts human health [22,23,24,25].

### 5.2. Diverse Policy-Related Studies

In various policy-related studies, social scientists investigate power relations between polluters, communities, decision-makers, scientific experts, activists, and different levels of government. While these studies require a sufficient level of EH knowledge, scholars are more focused on accounting for the intricacies of social factors than EH factors. Topic areas include studies of military contamination, precautionary consumption, and environmental health issues in natural resources such as fracking and water.

#### 5.2.1. Social Discovery of Military Contamination

Brown [26] and Shriver [27,28] have contributed extensive research into the social discovery of Gulf War-related illness and associated controversies over veterans’ environmental exposures. This scholarship has revealed the complex entanglements between EH issues and military practices and technologies and builds on prior accounts of military contamination such as Vietnam veterans’ suffering the health consequences of Agent Orange and “atomic veterans” exposed to radiation.

Brown and colleagues examine why studying the causes of Gulf War Illnesses—a complex of physical and neurological symptoms experienced by veterans of the 1990–1991 Gulf War—was far more complex than simply “following the molecule,” or chemical, to pinpoint a precise source of harm. They find that Gulf War veterans’ illness claims were undermined by incomplete data, scientific uncertainty, multiple exposures, and political controversy [26]. Similarly, Shriver and colleagues investigate Gulf War veteran efforts to construct and legitimize an environmental illness frame, along with the U.S. government’s subsequent refusal to recognize that frame despite complaints from over 100,000 individuals [27,28]. Against the backdrop of current and future 21st-century U.S. militarism, such analyses have enduring relevance to human and environmental health.

#### 5.2.2. Precautionary Consumption

Szasz [29] and MacKendrick [30] explore the rise of an individualized ethic of environmental health hazard protection. Szasz describes “inverted quarantine” as an individualized response to a collective threat, in which people practice self-isolation and consume goods they believe to be “safe”. For example, people buy organic food because they are concerned about pesticide exposure, rather than advocating for policies that would restrict the use of hazardous pesticides; this leaves the current model of industrial agriculture unchallenged and may do little to reduce chemical exposure, particularly for agricultural workers [31]. Szasz notes that inverted quarantine is largely ineffective given the ubiquity of contemporary environmental health threats. Instead, it offers a false sense of safety but little real protection against chemical hazards and leads to diminished public concern about EH issues that undermine the likelihood and perceived urgency of collective policy solutions [29]. Similarly, MacKendrick examines the lived experience of precautionary consumption as a gendered phenomenon in which women, especially mothers, internalize the precautionary ethic as an extension of their maternal responsibility. With environmental justice in mind, MacKendrick describes how precautionary consumption exacerbates already existing inequalities along the axes of race, gender, class, occupation, and geography, thus reinforcing and reproducing environmental and health inequalities. Instead of individualized approaches, MacKendrick advocates for policy recommendations related to chemical manufacturing and industrial agriculture, and for greater transparency in industry science and lobbying activity [30].

#### 5.2.3. Studies of Environmental Health Issues in Natural Resources

Social scientists from various disciplines have engaged in policy-relevant research on natural resource topics. In response to a fly-over ban designed to prevent public documentation of the BP Deep Water Horizon spill, anthropologist Sara Wylie collaborated on a novel community-monitoring project of balloon mapping that affixed digital cameras to tethered balloons to capture the dispersion of oil across the coast [32]. This led to the founding of the Public Laboratory for Open Science and Technology, a non-profit organization dedicated to the development of low cost, DIY community environmental monitoring tools [33].

Much social science research on water issues has been conducted through the Urban Water Innovation Network (UWIN), a transdisciplinary consortium of 15 academic institutions across the U.S. One example is UWIN’s Social Equity and Environmental Justice Lab, housed at Northeastern University and directed by sociologist Sharon Harlan. UWIN foregrounds community perspectives on the root causes of water inequality, describes local community members’ visions of what “sustainable” and “just” water systems look like in practice, and emphasizes the ways in which low-income communities of color are disproportionately saddled with water-related problems such as unaffordability, proximity to polluting facilities, and higher contamination [34].

Another example of social scientists’ critical engagement with environmental health issues is the Environmental Data and Governance Initiative (EDGI), a decentralized network of 170+ academics, professionals, and volunteers that formed following the 2016 presidential election [35]. EDGI studies governance and research practices and information dissemination by federal environmental agencies as part of a broader movement towards Environmental Data Justice [36]. They do this by monitoring federal websites, archiving environmental data, and interviewing current and former employees of federal environmental agencies.

##### 5.2.4. “Chemical Legacy” Studies

What we term “chemical legacy” studies constitute another form of SS-EH that focuses generally on chemicals of concern rather than on specific sites of contamination. Social scientists conducting this form of research must develop sufficient interactional expertise on a topic to understand the relevant environmental health research and policy issues and controversies [37], often through engagement with EH scientists and regulators. Historians Gerald Markowitz and David Rosner have written about lead and polyvinyl chloride (PVC), drawing on huge volumes of documentation they received through working on litigation. Their book *Deceit and Denial* examines corporate secrecy and duplicity across multiple industries, identifying impacts to working-class, poor, largely black workforces and communities [38]. One significant outcome of their work was building “Project Toxic Docs”, a website starting with those documents and then including more documents on what they describe as “millions of pages of previously classified documents on industrial poisons” (www.toxicdocs.org). It includes material on Polychlorinated biphenyls (PCBs), asbestos, silica, and many other chemicals, and our SSEHRI team contributed an EPA dossier on per- and polyfluoroalkyl substances (PFAS) as well.

Other social scientists have focused on particular chemicals or chemical classes. Sarah Vogel’s work on the toxic plasticizer bisphenol-A (BPA), begun while she was a visitor at the Chemical Heritage Foundation (now the Science History Institute), led her to a career in environmental health advocacy and science [39]. Nancy Langston’s *Toxic Bodies: Hormone Disruptors and the Legacy of DES*, is a comprehensive study of the discovery of diethylstilbestrol (DES), a synthetic estrogen mistakenly prescribed to women to prevent miscarriage that increased risks for many reproductive health problems, including a rare form of vaginal cancer in the daughters of women who had taken DES during pregnancy [40].

Alissa Cordner and Phil Brown studied flame retardants with intensive engagement with scientists, learning about chemical policy and science at EPA offices and academic centers, and engaging with state regulators to create significant policy changes. In addition to numerous social science articles and a book [41], they published studies of environmental health research trajectories, environmental and health policy, and occupational exposure reduction in interdisciplinary health and environmental science journals [42,43,44]. Lauren Richter, Alissa Cordner, and Phil Brown currently conduct similar work on PFAS compounds, learning much about the science and policy through deep involvement with scientists and government agencies, and publishing in both social science and environmental health and science journals [45,46,47].

In addition to being knowledgeable about the science, these “chemical legacy” scholars provide technical capacity in these fields through government testimony, advisory board participation, and scientific webinar presentations [47]. Rosner and Markowitz, for example, were called as witnesses in Rhode Island’s nationally significant lawsuit against major paint manufacturers. In our work on PFAS, we have extensive engagement with affected communities, including joint research projects and government policy action in collaboration with residents and EH scientists. We discuss this work in greater detail below.

## 6. Social Science-Environmental Health Collaborations

Mainstream EH science rarely discusses the political and economic factors that shape the production and interpretation of knowledge, including regulatory inadequacy, corporate manipulation of science and science-policy, and power differentials between researchers and participants. Yet many researchers are critical of traditional science and realize the need for their work to contribute to meaningful change efforts. This then leads to the third major grouping, what we term “social science-environmental health collaborations”. In these forms, social scientists conduct research directly on environmental health exposures and/or effects and collaborate formally or informally with environmental health scientists. When this research involves collaboration with impacted communities (e.g., CBPR partnerships), additional challenges are worth noting: communities and researchers may have different ideas and preferences in terms of project goals, timelines, control over data, consequences of identifying contamination, and communication and use of findings [48]. However, collaborative research offers additional benefits to both researchers and impacted communities through information sharing, training and research experiences, empowerment of impacted communities, improved recruitment of participants and quality of collected data, and increased research capacity [1,3].

### 6.1. Civic Science

Civic (or citizen) science refers to many forms of science conducted by people without official scientific credentials and is generally seen as a sign of the growing democratization of scientific practice [49]. The term “civic science” is increasingly used rather than “citizen science” because of the problematic use of the word citizen regarding immigrants, Native Americans, Alaska Natives, and First Nations. Additionally, the “civic” component denotes active political participation. In EH, this research often involves exposure scientists, sociologists, anthropologists, and STS scholars working alongside the exposed community and researchers with EH training. These investigations are often, though not always, community led, with support from trusted scientific and technical allies, and focused on the discovery or documentation of local exposures, an expansive understanding of effects, and accurate, detailed measurement, as well as social and political work towards remediation, redress, or other desired outcomes.

Examples of this type of scholarship abound. Public Lab, mentioned earlier, develops low-cost sensors and validates them against more established, technical methods. Edmund Seto at the University of Washington works with community groups, placing low-cost sensors next to EPA air monitors, to show how they are as capable of detecting air pollution as the official and more technical devices. Sara Wylie at Northeastern University works with community groups to test low-cost sensors for hydrogen sulfide, a potent neurotoxin and exposure of concern for fracking communities. These low-cost sensors equip communities with tools to easily and cheaply monitor the oil and gas infrastructure in their localities. These studies share a commitment to facilitating rapid response to community exposures through the provision of technical expertise, coupled with public and scientific validation of community knowledge of exposures. They also generally share a deliberate effort to give back to their community partners by developing public repositories of their core data and reports, along with efforts to communicate those data for consumption in popular media.

Civic science challenges the traditional hierarchy between researchers and participants or communities. Coburn focuses on the contestation over expertise and knowledge production inherent within this model, highlighting how deliberation and engagement are “necessary for the co-production of expertise” [50] (p. 41). Such an approach requires an abundance of humility and empathy on the part of the research team to recognize the legitimacy of the epistemological contributions of the affected community, whose knowledge is based in the lived experience of the associated exposures with their own bodies often acting as the primary sensors. Kimura and Kinchy emphasize the importance of viewing the affected community not just as participants or subjects, but as co-producers of knowledge and invested partners in associated social, political, and economic advocacy efforts [49]. This is a fundamental departure from other models of scientific inquiry that foreground the importance of peer-reviewable scientific validity at the expense of a direct, mutually beneficial relationship between researchers and their community partners. Scientific validity remains important, of course, but the first peer review in the civic science model comes from the affected community.

### 6.2. Multiple Researcher, Multiple Institution Collaborations

Many of these are academic-community partnership projects funded by the NIEHS’ earlier or current EJ, CBPR, and Research to Action (RTA) programs. Research and practice in these collaborations have been central to advancing knowledge and public health practice in community discovery of toxic contamination, cumulative impacts of many forms of environmental hazards and land-use practices, stress-environmental interactions, social support and networks, community organizing, and environmental health literacy [51,52]. Examples include asthma intervention and reduction and food security in Los Angeles public schools; occupational health for Chinese restaurant workers, Vietnamese floor sanders, and Cape Verdean housecleaners; Alaska Natives and military toxics; hog farms in black rural areas of North Carolina; Navajo struggles around uranium mines; and contaminated subsistence fish and the Yakama Tribe. More recent RTA projects include using low-cost air monitors to track air pollutant exposures and improve the health of residents in Imperial County, California; reducing mercury exposure to rural Oklahoma subsistence fishers; improving the health of Houston’s communities that border metal recycling facilities; and transforming fish consumption advice for Native American tribal communities living in and around the Great Lakes. Our PFAS-Research and Action for Community Health (PFAS-REACH) project, funded through the RTA program, started work in 2018 to assess immunotoxicity impacts of exposure to PFAS and develop educational materials for affected communities and other stakeholders, all with high levels of engagement from multiple community partners.

Our work on report-back of personal exposure data (from both biological and environmental sampling) to participants is an especially unique SS-EH collaboration. In some of Silent Spring Institute’s initial exposure and biomonitoring projects measuring personal and household exposures to carcinogens in the home environment, participants asked when they would get their data reported to them. The researchers immediately understood the importance of this and initiated a long series of projects to do such report-back, adding sociologists to their research team. The researchers reflexively engaged with the community and research participants to (1) understand the individual and collective needs of participants related to their environmental health data; (2) understand how taking part in research and receiving data influences the creation of shared definitions of exposure; (3) investigate how personal and collective histories influence the interpretation of data; and (4) to understand generally how receiving environmental health data influences participants personally and politically [53].

This collaboration has continued for 15 years with NIEHS and National Science Foundation (NSF) funding and to our knowledge is the longest SS-EH collaboration in existence. From initially doing and analyzing our report-back process, we expanded to studying how other researchers choose to report back or not, how Institutional Review Boards (IRBs) approved or rejected such approaches, and how study participants experienced the process and used the data at personal, community, and societal levels. We then studied data privacy issues that were raised by an EPA request to share de-identified data that we thought might be re-identifiable by using housing or other characteristics. Out of this research, our team published over 20 articles and handbooks on report-back and data privacy, some which are cited in this article.

The integration of social science in Silent Spring Institute’s Household Exposure Study and subsequent projects has created new concepts such as the “research right-to-know” [54], “exposure experience” [55], and “politicized collective illness identity” [56]. This research has redefined and restructured environmental exposure studies, increased public understanding, furthered environmental health literacy, and facilitated community empowerment and mutual trust and respect between researchers and communities. This work has also contributed to report-back becoming a more acceptable and more widely used practice in government and academic research. Prompted by our work, NIEHS dedicated its 2018 Partnerships in Environmental Public Health conference to the topic of report-back.

An additional example of SS-EH collaboration comes from the research conducted by Sabrina McCormick and her collaborators into the health effects associated with exposure to increasingly extreme heat. In partnership with WE ACT for Environmental Justice in Washington, DC, McCormick and her collaborators conducted in-depth interviews and policy analysis to examine preparations for expected extreme heat events associated with climate change in four major U.S. cities [57]. The research team brought to bear expertise in sociology, public health, epidemiology, behavioral health, and environmental science to determine what factors influenced the capacity and will of a city to protect public health from extreme heat events, providing feedback to those officials and the public to help inform future development of better practices and policies.

Similarly, Jonathan London co-directs the University of California-Davis Environmental Health Science Core Center’ CEC. The CEC fosters a culture of collaboration among environmental health scientists, social scientists, community organizations, environmental justice experts, and environmental health-related public agencies to create and sustain multi-directional partnerships for environmental health research that are responsive to the environmental health needs and concerns of the most vulnerable communities in California’s Central Valley. The CEC’s social scientist staff provide a critical perspective on the structures and processes that shape environmental and health disparities, and a significant portion of their funding program to support community-engaged environmental health sciences research goes to social scientists working on community-initiated projects.

### 6.3. University Centers and Training Programs

Interdisciplinary centers and multi-level training programs have been central to the success of SS-EH research. In this section, we draw on our own experience with two multidisciplinary research groups, CIRG and SSEHRI, both of which embody the SS-EH collaborative model. While some of these groups’ work has been mentioned before, here we offer a deeper look into the organizational structure of those centers, their training programs, and their conferences.

The university is a critical site for the development, location, and support of SS-EH collaborations. Practically, the university provides both institutional space for SS-EH research centers, and administrative apparatuses to act as funding agents for any awards provided to the center and to manage associated students and faculty. Through this mutually beneficial relationship, the research center makes use of the university’s facilities and administrative capacities and provides funding to the university in support of those provisions. Though an SS-EH research center certainly could exist unanchored from a university, the symbiotic relationship helps the center take advantage of institutional resources that would not likely be available to it otherwise. 

This work is likely easier to accomplish at large research-oriented universities, which have more established and well-resourced institutional systems for research support such as larger grants offices and support for course buy-outs. However, institutional affiliation at smaller universities or even teaching-focused colleges does not preclude involvement and even leadership in SS-EH collaborations. Alissa Cordner is Associate Professor of Sociology at Whitman College, a small liberal arts college in rural Washington State. Through funding arrangements and collaborations with faculty at larger institutions, she serves as Co-Principle Investigator and research lab leader for SSEHRI’s PFAS research. As another example, anthropologist David Bond is Associate Director of the Center for the Advancement of Public Action at Bennington College, a small liberal arts college in Vermont. He helped launch a SS-EH collaboration to identify health impacts of PFAS exposure in Bennington, VT and the nearby community of Hoosick Falls, NY. These examples show that researchers at smaller academic institutions can leverage collaborations and location-specific resources to effectively pursue SS-EH collaborations.

Even more important than resources, however, is the intellectual and cultural value of the university as a site with high levels of intellectual capital, passionate students and faculty, established connections with surrounding communities, and deep personal and professional networks. For SS-EH research institutes in particular, the university also provides an opportunity to develop the interdisciplinary connections that facilitate concrete outcomes for impacted communities in response to complex problems. Both SSEHRI and CIRG stand as examples of the benefits of the critical role the university can play supporting SS-EH research centers.

When CIRG started at Brown University in 1999, it was centered on a project funded by the Robert Wood Johnson Foundation and NSF that examined disputes over environmental causation of asthma, breast cancer, and Gulf War illnesses, as well as exposure reduction approaches using the precautionary principle and toxics use reduction [58,59]. With additional funding from NIEHS and NSF, CIRG expanded to include research on labor-environment coalitions, biomonitoring and household exposure, report-back of environmental data to participants, Native American environmental health organizing, local organizing around toxic waste and cleanup, involvement with a statewide EJ organization, school siting on contaminated land, and the social and scientific discovery of flame retardant chemicals. Starting with its initial focus on contested illnesses, CIRG partnered with several community partners, including Silent Spring Institute. Much of the SS-EH research discussed in this article arose out of the early work of CIRG.

SSEHRI was formed at Northeastern University in 2012. SSEHRI currently has affiliated faculty at Whitman College, University of Vermont, and Brandeis University, along with numerous post-docs, graduate students, and undergraduates. Its mission is “to conduct social science-oriented research, teaching, community engagement, and policy work in the area of environmental health”, and to act as “a hub for collaborative environmental health learning and interest” [60] (p. 5). SSEHRI’s work has been supported by the NIEHS, NSF, the JPB Foundation, and others to examine the social and scientific discovery of toxic chemicals; continue work on exposure studies and report-back; examine data privacy of environmental health and civic science projects that involve community monitoring; and improve water access and equity in impacted communities.

In partnership with the Silent Spring Institute and several grassroots organizations, SSEHRI engages in community-based SS-EH collaborations in areas of concern to impacted communities. At the same time, SSEHRI provides support for faculty research projects as well as for students and post-docs, helping to create and continually reproduce a cohort of researchers and collaborators from diverse academic traditions, including sociology, environmental and environmental health science, and science and technology studies.

### 6.4. Training Programs

Disciplinary silos, trans-disciplinary communication challenges, and funding limitations mean that SS-EH integration is not an easy or natural research process. Instead, researchers must consciously develop it by linking the actual practice of collaborative research with more formalized training programs. We have pursued formal and informal training programs in our work. For example, our NIEHS-funded SRPs and Children’s Environmental Health Centers provide experience for graduate students and post-docs in multi-project centers that value contributions from social science. Participation in this type of center involves hands-on training in community engagement, scientific writing, and research translation.

A more formalized training model can be found in the NSF and NIEHS Training Program Grants. Here we describe our own experience. Building on the collaboration between Silent Spring Institute and Brown University (later at Northeastern University), we launched an NSF Science and Technology Studies Training Program, “New Directions in Environmental Ethics: Emerging Contaminants, Emerging Technologies, and Beyond”, that ran from 2012 to 2016. It supported sociology doctoral students and post-docs from sociology, anthropology, and STS. This laid the foundation for a more extensive, five-year NIEHS T32 Training Grant, “Transdisciplinary Training at the Intersection of Environmental Health and Social Science”, that began at Northeastern in 2015. The T32 program requires a complex and multi-disciplinary training infrastructure, including academic mentorship, methods training, and community engagement. Our Training Grant prepares Northeastern’s sociology doctoral students (including co-authors Grace Poudrier and Jesse DiValli) and environmental health post-docs to work in environmental health and the social sciences, to build connections across disciplines, and to work collaboratively to address complex socio-environmental problems. Students take coursework in environmental health and environmental sociology to understand the complex entanglement of cultural, political, historical, environmental, and genetic factors that shape health. They also engage in dedicated seminars and receive methodological training in community-based participatory research for environmental health, environmental justice, and civic science. Sociology doctoral students work in one of the three lab groups led by SSEHRI faculty: PFAS Project, Water Equity, or Low-cost Community Monitoring. They also take part in research projects at the Silent Spring Institute. Post-docs, coming from environmental health doctoral programs, are based largely at Silent Spring, but also take part in one of the SSEHRI labs and mentor doctoral students. Separate meetings of the Training Program provide skill-based learning on topics ranging from air sensors and exposure science to op-ed writing and data security. Trainees accumulate extensive experience in oral and written presentation and give guest lectures in related classes taught by SSEHRI faculty.

### 6.5. Conferences

Conferences provide essential forums to discuss SS-EH, to make visible existing collaborations, and to bring people together that may not know of each other’s work. SS-EH focused conferences can involve academic participants from a range of disciplines or can be focused on a topic and extend far beyond academia to include a broad range of relevant stakeholders.

As an example of a transdisciplinary academic conference that brought together previously disparate researchers and made visible existing collaborations, Brown University’s SRP and NIEHS organized a 2012 workshop in Providence, RI. The workshop brought together researchers from many disciplines and institutions to examine how social science research could advance life and physical science research aimed at characterizing the human health risks of hazardous waste sites and improving cleanup plans. Following the typology presented in this article, this workshop emphasized both the social science of environmental health and environmental health research influenced by social science.

As another example of the transdisciplinary conference model, in 2015 SSEHRI and Northeastern’s SRP center hosted the first Social Science-Environmental Health Interdisciplinary Collaborations Conference [2]. Over 100 attendees from a range of social science and environmental health disciplines, advocacy organizations, and government agencies participated, all working on environmental health and social sciences. The conference allowed them to come together to foster collaboration and better address complex human-environmental health issues.

Conferences supporting SS-EH collaborations can also be explicit in breaking out of academic boundaries by focusing on a specific issue rather than a particular discipline or method. For example, the 2017 National PFAS Conference was organized by SSEHRI in collaboration with the Silent Spring Institute, Testing for Pease, and the Toxics Action Center. (The 2nd National PFAS Conference took place in June 2019). It brought together 180 attendees including scientists, community advocates, government officials, state legislators, journalists, environmental advocates, lawyers, and students, all of whom worked on PFAS-related science, regulation, and advocacy. Panels included both social scientists and natural scientists, including presentations by researchers doing SS-EH collaborative research. The conference was extremely successful at establishing new relationships and strengthening existing ones. For example, the National PFAS Coalition of impacted communities formed as a result of the conference [61]. Testing for Pease’s central role in the conference laid the foundation for their collaboration with the Silent Spring Institute and SSEHRI in the REACH project as well as a foundation grant for water testing. Contacts with researchers across the country led SSEHRI and the Silent Spring Institute to develop a proposal for a national-level multi-pronged PFAS research center.

## 7. Conclusions

The typology outlined in this article identifies three major forms of SS-EH research: social science influencing EH, EH influencing social science, and SS-EH collaborations. Our focus in this article has largely been on our own work, most centrally on the three decades of community-engaged research by Phil Brown and the numerous collaborations and programs associated with his research centers. In describing how researchers are engaging in this important work, we emphasized both the challenges and benefits of doing SS-EH research, and furthermore discussed how training programs and conferences can provide the necessary resources and spaces for such collaborations. Identifying different forms of SS-EH allows researchers to consider new potential forms of SS-EH for their research, along with the resources, skill-sets, and practices central to different ways of doing collaborative research.

SS-EH work is often challenging, can be extremely time-consuming, and involves additional resources and skill-sets not central to academic training. Scholars seeking to take a route similar to ours should investigate the reputation of their university or institution in the local communities with whom they hope to work. Many community members and groups have experience with universities’ disregard for communities, sometimes including trauma from land expansion, unethical research practices, or other actions. Researchers may thus find they need to work on healing through engaged work and relationship-building. We encourage researchers from all disciplines to join in forming SS-EH collaborations. However, even for those who do not, we believe there is much to learn from the research that we describe.

Conducting SS-EH research demands constant reflexivity on the part of the researcher regarding ethical guidelines, decision-making principles, and relationships between researchers and participants related to their assumptions, methods, analyses, and applications. That is both an ethical approach and a method by which we continually improve the work we do [3]. We would also note that our SS-EH research draws on our training as critical environmental sociologists to critique the political and economic factors that create conditions of environmental health problems and inequalities. Thus, our research explicitly takes a precautionary approach to its goal of improving environmental and public health.

We have argued that full-fledged collaborations between social scientists and environmental health scientists harbor the greatest potential to improve public and environmental health through the fullest range of possible research approaches. Future research should include a systematic analysis of publications resulting from social science-environmental health collaborations. This research should incorporate interviews with researchers who are cited here, as well as others identified through a sampling of the NIEHS centers and programs that have historically included social scientists. A project of this nature would be well positioned to elaborate on the challenges, obstacles, and successes of SS-EH. We also encourage researchers engaged in all three forms to spend time analyzing their type of scholarship and social action to gain a reflexive understanding of their efforts. In addition to individually reflexive analysis, there is a need for reflexive organizational ethnographies of SS-EH projects, which can help group participants to pause and analyze their collective endeavor while providing a look at whole-organizational structure (c.f. Vera and colleagues’ autoethnography of EDGI [62]).
